# Climate Change and Dispersal Ability Jointly Affects the Future Distribution of Crocodile Lizards

**DOI:** 10.3390/ani12202731

**Published:** 2022-10-11

**Authors:** Xiao-Li Zhang, Facundo Alvarez, Martin J. Whiting, Xu-Dong Qin, Ze-Ning Chen, Zheng-Jun Wu

**Affiliations:** 1 Key Laboratory of Ecology of Rare and Endangered Species and Environmental Protection, Guangxi Normal University, Ministry of Education, Guilin 541004, China; 2 Guangxi Key Laboratory of Rare and Endangered Animal Ecology, Guangxi Normal University, Guilin 541004, China; 3 Programa de Pós-Graduação em Ecologia e Conservação, Campus Nova Xavantina, Universidade do Estado de Mato Grosso, Nova Xavantina 78200-000, Brazil; 4 School of Natural Sciences, Macquarie University, Sydney 2109, Australia; 5 Guangxi Daguishan Crocodile Lizard National Nature Reserve, Hezhou 542800, China

**Keywords:** climate change, dispersal, Maxent, MigClim, *Shinisaurus crocodilurus*, Zonation

## Abstract

**Simple Summary:**

Climate change is one of the most significant global challenges we face this century, and all species will likely be affected by it. Climate change may cause organisms to advance their phenology, delay reproduction, shift their ranges northwards, or even reduce their range. Reptiles, as typical ectotherms, are more sensitive to climate change, mainly in the form of range shifts. The crocodile lizard (*Shinisaurus crocodilurus* Ahl, 1930) belongs to a monophyletic family and genus and is a relict species from the late Quaternary glacial period. Given its unique evolutionary history, it arguably has greater conservation value. The overall population size of this species is decreasing due to habitat destruction and over-exploitation (e.g., traditional medicine, food, and pet trade). This study evaluated the effect of climate change and dispersal capacity on the potential distribution range of crocodile lizards. Temperature and precipitation interact to affect the distribution of crocodile lizards. Historical climate has seen a large expansion in the range of the crocodile lizard. However, in a future global warming scenario, the potential range of crocodile lizards will continue to shrink. The same results were obtained for different migration scenarios. However, to better predict future changes to the distribution range of the crocodile lizard we need more fieldwork and ground truthing. All we can do currently is try to protect the lizard and its habitat to ensure their continued survival.

**Abstract:**

Crocodile lizards (*Shinisaurus crocodilurus*) are an endangered, ‘living fossil’ reptile from a monophyletic family and therefore, a high priority for conservation. We constructed climatic models to evaluate the potential impact of climate change on the distribution of crocodile lizards for the period 2000 to 2100 and determined the key environmental factors that affect the dispersal of this endangered species. For the construction of climatic models, we used 985 presence-only data points and 6 predictor variables which showed excellent performance (AUC = 0.974). The three top-ranked factors predicting crocodile lizard distribution were precipitation of the wettest month (bio13, 37.1%), precipitation of the coldest quarter (bio19, 17.9%), and temperature seasonality (bio4, 14.3%). Crocodile lizards were, just as they are now, widely distributed in the north of Guangdong Province in China and Quảng Ninh Province in Vietnam at the last glacial maximum (LGM). Since the LGM, there has been an increase in suitable habitats, particularly in east-central Guangxi Province, China. Under future global warming scenarios, the potential habitat for crocodile lizards is expected to decrease significantly in the next 100 years. Under the most optimistic scenario, only 7.35% to 6.54% of suitable habitat will remain, and under the worst climatic scenario, only 8.34% to 0.86% of suitable habitat will remain. Models for no dispersal and limited dispersal showed that all crocodile lizards would lose habitat as temperatures increase. Our work contributes to an increased understanding of the current and future spatial distribution of the species, supporting practical management and conservation plans.

## 1. Introduction

Climate change is one of the most significant global challenges we face this century, and all species will likely be affected by it. The vulnerability of an organism to environmental change depends on its history and exposure to environmental change, its sensitivity to this change, and its ability to recover or adapt to it [1]. Climate change is cited as a cause for the decline in 19% of threatened or near-threatened species listings by the International Union for the Conservation of Nature and Natural Resources (IUCN) [2]. Recent assessments show that at least 40% of amphibian species, 30% of reef-forming coral species, and one-third of marine mammal species are threatened with extinction [3]. Lepidosauria, which includes lizards, snakes, amphisbaenians, and the tuatara, is the most diverse clade of extant reptiles in terms of the number of species and morphological, physiological, and ecological characteristics [4]. Because lepidosaurs are ectothermic, several of their life history traits are likely to be influenced by environmental temperature variation [5], making them excellent models to evaluate the potential effects of climate change on terrestrial ectotherms [6]. In order to mitigate the negative effects of climate change on species, conservation strategies should be refined by first modeling species distributions to identify to what extent they could be influenced by future climate change [7].

Chapter IV of the IPCC Sixth Assessment Report (AR6) Working Group I Report evaluates the future global climate estimates of global regions and the climate system [8]. The assessments show that over the next two decades, the global mean surface air temperature will reach 1.5 °C or even beyond it, and mean-state and variability of precipitation would increase as well, but varying with seasons and regions [9]. Climate change and related impacts are the main and common causes of disturbance mechanisms that lead to the emergence of new species combinations, changes in species ranges, local extinctions, reduction in biodiversity, loss of ecosystem resilience, and previously unobserved disturbance mechanisms [10,11,12,13]. Global warming can affect sex ratios through a range of mechanisms [14,15]. High temperatures can distort sex ratios through three main mechanisms, namely sex-biased heat tolerance, temperature-dependent sex determination (TSD), and temperature-induced sex reversal [14]. Recent data suggest that climate change may be causing extreme sex ratio distortions in TSD species. For example, up to 99% of sea turtles have been reported in warmer areas [16], and single-sex populations are projected for the near future in other TSD reptiles including lizards, crocodilians, and the tuatara [17]. In many cases, global climate change can trigger significant loss of habitable areas [18,19]. This phenomenon has been pointed out as the cause of several extinction events [20,21]. However, if the portions of a species’ occupied range have physical characteristics that buffer the rate of contemporary climate change, then the area where sustainable replenishment is occurring may behave as an in situ climate refuge, or move to a nearby range (relocated refuge) [22,23,24]. For some species, the current rate of change, combined with habitat loss and fragmentation, will exceed their ability to adapt or disperse, and migration to climatically suitable habitats is an important option for maintaining viable wild populations [25,26]. Organisms change their geographical distribution through natural dispersal/migration or forced dispersal/migration because of human and environmental pressure to track preferred environmental conditions [27,28,29,30,31]. Globally, species distribution has shifted to higher elevations and latitudes at different rates [13]. As ectotherms, reptiles are sensitive to the effects of environmental temperature, especially given that their environments (and thus, body temperatures) typically experience dramatic thermal fluctuations daily, seasonally, and stochastically [32,33]. Thus, behavioral plasticity and the ability to move in search of thermally suitable habitats are important strategies used to buffer climatic fluctuations [5].

One of the current approaches to quantifying the future distribution of species under climate change is the application of environmental niche models (ENMs), also known as species distribution models (SDMs), which consist of a set of algorithms and general data management procedures that seek to model the potential or realized geographic distributions of species, typically based on their known occurrences and several environmental predictors [34]. ENMs have been used to investigate the factors driving species distribution (e.g., phylogeography), and estimate the dynamic changes of climate niches [35,36]. In particular, ENMs have been employed to forecast species responses to climate change, to search for new populations of rare species, and to predict the impacts of land use on species distributions (e.g., [35,37,38]). Despite the widespread use of ENMs, the reliability of their results depends on the characteristics and quality of the occurrence data used to train them [39], and the limitations of the main biodiversity data used for modeling may also limit its utility [40,41,42]. However, under climate change one of the most implausible assumptions of conventional ENMs is that the species distribution is in equilibrium with climate, i.e., species will immediately react to the changing climate by shifting, expanding, or contracting their ranges [43]. This assumption is highly improbable, as it does not consider species’ biotic responses and dispersal or migration constraints [44,45], thus resulting in under- or overestimation of species distributions. Hence, the migration capacity of a species is a crucial factor for predicting its distribution under climate change, and various modeling approaches incorporate more realistic migration into ENMs [46].

The crocodile lizard (*Shinisaurus crocodilurus* Ahl, 1930) belongs to a monophyletic family and genus and is a relict species from the late Quaternary glacial period [47]. Given its unique evolutionary history, it arguably has added value [47]. The overall population size of this species is decreasing due to habitat destruction and over-exploitation (e.g., traditional medicine, food, and pet trade) [48,49]. This species is currently listed as endangered on the International Union for Conservation of Nature (IUCN) Red List [50]. The crocodile lizard is a diurnal lizard that spends 69% of its total active behavior time in a sedentary position [51,52]. They require constantly cool water and adjacent terrestrial areas [51,52,53]. Additionally, the occupied temperature niche of crocodile lizards is narrower compared with the environmental fundamental niche, with the temperatures of the animal being generally cooler compared with environmental temperatures [53]. Chinese crocodile lizards hibernate from October to April and initiate hibernation at temperatures between 8 and 11 °C while Vietnamese crocodile lizards initiate hibernation at mean temperatures of 17.5 °C in December and start becoming active without a pronounced increase in temperature in March [53,54,55]. Reptiles are particularly vulnerable to heat stress because they have to regulate their body temperature behaviorally [56,57]. Without sufficient opportunity for behavioral or physiological adaptations to increase resilience under climate change, population declines could herald local species extinctions, or even global extinctions [26,58,59]. Our study had two major objectives: (1) model (and predict) the distribution patterns of crocodile lizards under historical and current climate change scenarios; and (2) determine suitable habitat availability for this species under three possible dispersal scenarios (no dispersal, limited dispersal, and unrestricted dispersal).

## 2. Methods

### 2.1. Occurrence Data

**Figure 1:**
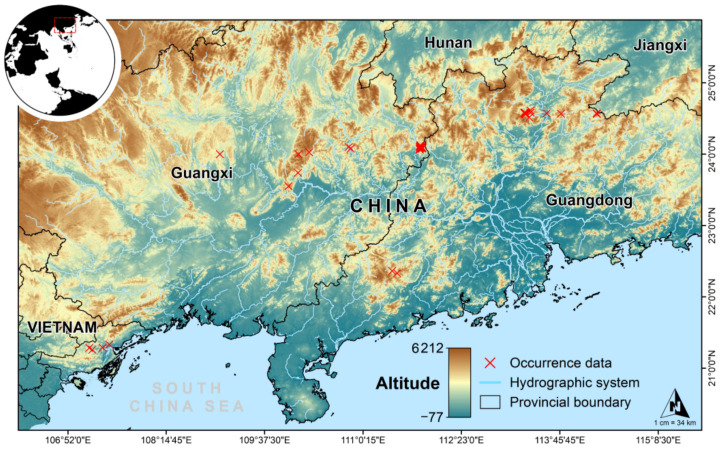
Geographic locations of *Shinisaurus crocodilurus* populations.

Crocodile lizards (*Shinisaurus crocodilurus*) are habitat specialists confined to densely vegetated rocky streams in cool forests found only in Guangxi and Guangdong provinces of southern China and Bắc Giang and Quảng Ninh provinces in northern Vietnam [48,49]. However, the historical distribution of the crocodile lizard was as far north as Jianghua County in Hunan Province and as far south as the Tropic of Cancer. Due to habitat destruction and subsequent fragmentation, the distribution range of this species is gradually shrinking [48]. We obtained a total of 1048 occurrences of the species from the following three sources: (a) fieldwork (830 records); (b) Global Biodiversity Information Facility records (GBIF, http://www.gbif.org/, accessed on 1 May 2021); and (c) published literature. We used Google Earth (http://ditu.google.cn/, accessed on 15 August 2021) to retrieve coordinates for occurrences that were missing geographical coordinates and discarded erroneous or duplicate records. To avoid spatial auto-correlation, we kept only one distribution point within each raster (1 km × 1 km). This left us with 985 occurrence records to be used in the modeling (Figure 1).

### 2.2. Climatic Variables

We used an initial 19 bioclimatic variables, which included the Last Glacial Maximum and current and future climate scenarios. All climatic variables were downloaded from the global climate data website (https://www.worldclim.org/, accessed on 22 September 2021). We used the Shared Socio-Economic Pathways (SSPs) of the International Coupled Model Comparison Program Phase 6 (CMIP6), which consists of five scenarios, from low to high anthropogenic radiative forcing values: SSP126, SSP245, SSP370, and SSP585 [60]. The first number after the SSP indicates the hypothetical shared socio-economic pathway, and the second number indicates the approximate global effective radiative forcing value by 2100 [9]. Temperature change varies between CMIPs, with CMIP6 being the ensemble that predicts stronger warming and can simulate global warming trends without climate policy intervention [60,61]. 

The environmental variables were resampled to a spatial resolution of 30-s (approximately 1 km above ground). Many environmental variables are spatially correlated, which might lead to the overfitting of predictions [62]. Therefore, Pearsonr’s correlation coefficients were used to examine correlations among the factors in ArcGIS 10.6. We only retained variables presenting low correlation values with all other variables (|Pearsonr| > 0.8) and eliminated the highly correlated (|Pearson r| ≥ 0.8) climatic variables [63]. Since crocodile lizards inhabit streams deep in dense forests and their activity increases during the rainy season, precipitation is the most critical limiting factor affecting crocodile activity [54,64,65]. Finally, we retained variables bio4, bio7, bio10, bio13, bio17, and bio19 (Table 1).

### 2.3. Modelling Procedures

This study was performed using the Maxent model, based on the maximum entropy theory that anything with maximum entropy is closest to its true state [66], which is a general-purpose machine learning method with a simple and precise mathematical formulation, and it has a number of aspects that make it well-suited for species distribution modeling [66]. Maxent version 3.4.1 [67] was used with the default parameters for a random seed, a regularization multiplier (1, included to reduce overfitting), maximum iterations (5000), a convergence threshold (0.00001), and the maximum number of background points (10,000 points that have not been recorded as presence). 

Seventy-five percent of crocodile lizard distribution data were randomly selected for model training, and the remaining 25% of the data were used for testing [68]. Twenty replications were performed, and the replicate runs were cross-validated to guarantee the accuracy of the model. We used the maximum training sensitivity plus a specificity (MTSS) threshold as recommended by Liu, Newell, and White [69] for model results output in logistic format, which is a cut-off threshold that precisely allows continuous predictions of suitability to be transformed into binary predictions of presences (1) and absences (0). At the same time, we reclassified the suitability into four classes: unsuitable habitat (<MTSS), minimally suitable habitat (MTSS–0.5), moderately suitable habitat (0.5–0.7), and highly suitable habitat (>0.7).

The AUC is a threshold-independent technique that is used to differentiate presence from absence to evaluate model performance [70]. The AUC value obtained can be interpreted as the estimated probability of the randomly selected grid cell in a correctly adjusted model, and AUC defines the success of the model with all possible thresholds [71]. To verify the accuracy of each model, we adopted the area under the curve (AUC) of the receiver operation curve (ROC), which was determined by the following criteria: poor (0–0.6), fair (0.6–0.7), good (0.7–0.8), very good (0.8–0.9), and excellent (0.9–1) [72].

### 2.4. Dispersal Analysis

We used a cellular automation model as implemented in the MigClim R package [73,74] to incorporate dispersal constraints of *S. crocodilurus* and predict potential distribution and invasion under future climatic scenarios for the period 2000–2100. The MigClim R package [74] is a function library for the open-source R software (version 4.0.4, Auckland University, Auckland, New Zealand) that enables the implementation of species-specific dispersal constraints into projections of species distribution models under environmental change and/or landscape fragmentation scenarios [74]. But it can equally well be used to simulate dispersal in stable environments and undisturbed landscapes (e.g., for modeling the potential spread of invasive species) [74].

We used MigClim to model the dispersal of *S. crocodilurus* over 100 years (2000–2100) under three dispersal scenarios: (1) unlimited dispersal, species can disperse to any suitable cell; (2) limited dispersal with significant barriers, species can disperse following the MigClim simulation, but are affected by dispersal barriers such as land cover classes, and (3) no dispersal [75]. To simulate dispersal under climate change, MigClim requires the following inputs: a map defining the species’ initial distribution, maps picturing landscape fragmentation (i.e., barriers to dispersal and permanent unfavorable locations), the species’ dispersal parameters, and a series of maps indicating how the distribution of potentially suitable habitats evolves as climate changes [73,74,75]. Combining the crocodile lizard habitat with our field surveys [49,64,65], we set the values for shrubs, rivers, reservoirs, and pools to be 0 as the dispersible area for the species in the land cover map of 2000 obtained from Globeland30 observations (http://www.globallandcover.com, accessed on 27 September 2021). All other modified and degraded land classes were given a value of 1 as a barrier to dispersal. At the same time, it was resampled to a spatial resolution consistent with the habitat suitability map as a diffusion barrier for the crocodile lizards. We set the MigClim models by the following settings (Table 2): rcThreshold = 500, envChgSteps = 5, dispSteps = 20, iniMatAge = 1, propaguleProd = c(0.01 0.08 0.5 0.92), lddFreq = 0.1, lddMinDist = 6, lddMaxDist = 15, replicateNb = 5 [73].

Finally, we converted the Habitat Suitability Index (HSI) of the habitat suitability map to an integer from 0–1000 as required by the model. All calculations and mapping were performed in ArcGIS 10.6 (ESRI, 2012) and R 4.0.4 (New Zealand, Auckland University).

### 2.5. Conservation Units

The Zonation is used to design wildlife reserves by minimizing the amount of space in a conservation area while meeting protection requirements [76]. The program also delimits regions that are priority protected areas (PPAs) according to real or potential species distributions [76]. Therefore, to calculate the priority areas for the conservation of our target species, we used the Zonation software (version 4.0.0: http://cbig.it.helsinki.fi/, accessed on 25 April 2022). We selected as input data the rasters: (1) Retention layer, potential natural vegetation raster (PNV, https://zenodo.org/, accessed on 25 April 2022); (2) Condition layer, human footprint raster (https://figshare.com/, accessed on 25 April 2022) and, (3) Feature map, the prediction obtained from the Maxent algorithm. To standardize the input data, we cut and fit the rasters to the same geographic extent and spatial resolution as the output prediction obtained from the Maxent algorithm. We included the retention layer following MigClim, first, because we used a species-specific data matrix [77,78]. Second, this model is less pessimistic as it is based on “management gain”: it assumes that the status of the species will increase in the absence of conservation. Finally, MigClim is more realistic because it balances habitat fragmentation/loss for species [77]. We included the conditioning layer so we could assess habitat loss/degradation. For this, we used an anthropic action indicator so that any negative value or those equal to zero represents degraded and unsuitable habitats for the species. We applied Core Area Zonation (CAZ) as an elimination rule, and to avoid conditioning the results to partial adjustments, we decided to keep the rest of the parameters by default: Boundary Length Penalty (BLP = 0) and Warp factor (200).

## 3. Results

### 3.1. Model Verification and Key Climatic Factors

**Figure 2:**
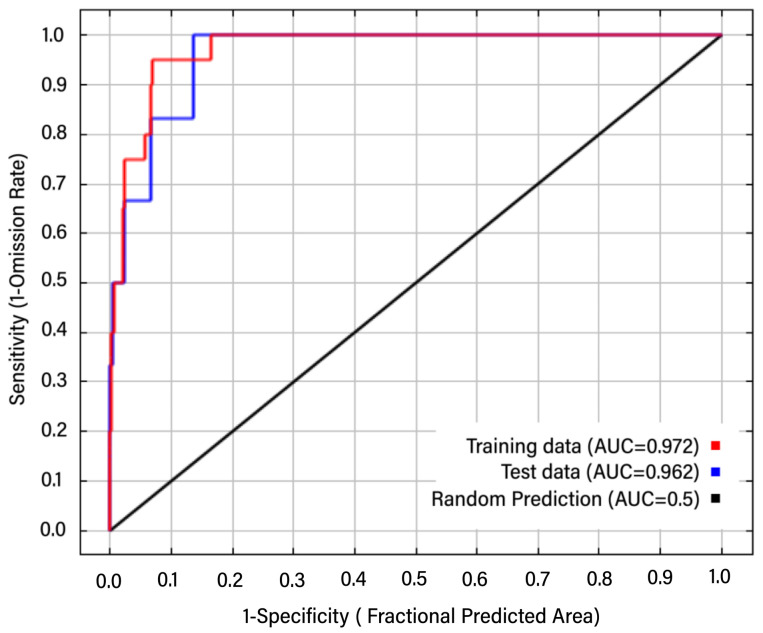
AUC value obtained from ROC analysis to test model predictions.

The AUC values of the Maxent models were higher than 0.9, indicating that our ENMs had excellent overall predictive ability (Figure 2). Among all variables, the five top-ranked factors were precipitation of the wettest month (bio13, 37.1%), precipitation of the coldest quarter (bio19, 17.9%), temperature seasonality (bio4, 14.3%), precipitation of the driest quarter (bio17, 10.7%), and annual temperature range (bio7, 10.4%), collectively, the predictive ability of the top five variables was 90.4% (Table 1).

### 3.2. LGM and Current Climate Scenario Analysis

**Figure 3:**
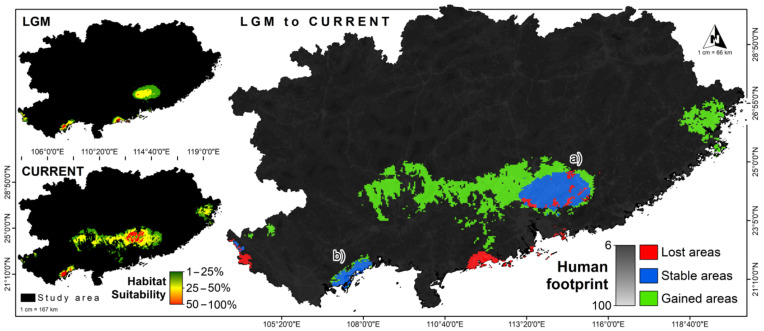
Potential distribution range of crocodile lizards under LGM and current climate scenario. a: The stable area of crocodile lizards distribution in China from LGM to current; b: The stable area of crocodile lizards distribution in Vietnam from LGM to current.

The distribution of *S. crocodilurus* under the LGM and the current climatic scenario is shown in Figure 3. A comparison of current and past model distributions suggests that the LGM suitable habitat has been lost in areas that include all the counties of Yangjiang in the southwest of Guangdong. At the same time, the comparison indicates that at the LGM crocodile lizards were, just as they are now, widely distributed in the north of Guangdong Province in China and Quảng Ninh Province in Vietnam. A comparison of current and past distribution models also shows an increase in habitat since the LGM, including areas in east-central Guangxi Province in China. Under the current climate scenario, high-quality habitats are mainly found in Shaoguan and Qingyuan of Guangdong Province and central Quảng Ninh Province, Vietnam. Medium-quality and low-quality habitats were concentrated in northern Guangxi (including Hezhou, Laibin, Guigang, and Hechi) and Shaoguan, Qingyuan, Heyuan, and Zhaoqing in Guangdong. The current potential distribution areas of crocodile lizards are consistent with our field surveys (all of the recorded crocodile lizards are distributed in the areas). These areas are mainly located in the middle of the mountains, where the vegetation consists of evergreen broad-leaved forests, secondary mixed scrub, bamboo forests, and mixed coniferous forests. The distribution areas are crisscrossed by streams and ravines, and the climate is temperate with simultaneous rain and heat.

### 3.3. Projected Future Change in Species Distributions

**Figure 4:**
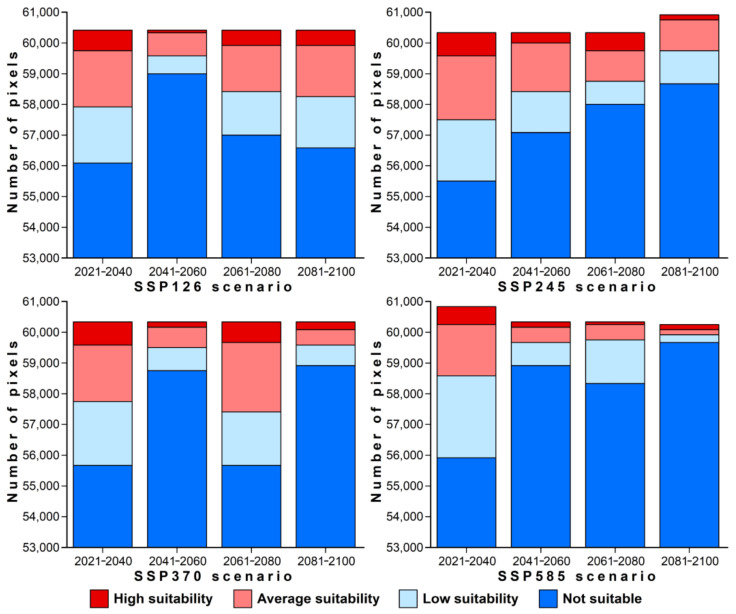
Pixels of crocodile lizard habitat under different climate scenarios.

**Figure 5:**
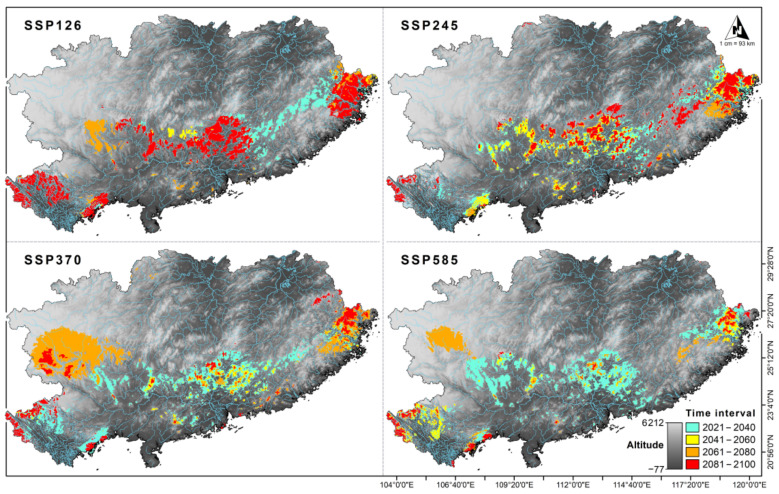
Potential distribution range of crocodile lizard under future climate scenarios.

With increasing radiative forcing, the suitable habitat for crocodile lizards will decrease. Therefore, under the climate change scenarios SSP126, SSP245, SSP370, and SSP585, the current habitats occupied by crocodile lizards are expected to decrease from 2021 to 2100 (Figure 4 and Figure 5). In the most optimistic climate scenario (SSP126), suitable habitat is expected to decrease to 2.34% from 2041 to 2060, and it would increase to 6.54% from 2081 to 2100. In this scenario, the medium habitat remained at about 2.50%, while the extreme value for high-quality habitat was 0.11%. Under climate scenarios with moderate radiative forcing (SSP245 and SSP370), high-quality habitat fluctuates periodically. Under SSP245, suitable habitat increased to 1.25% from 2021 to 2040, increased to 0.98% from 2061 to 2080, decreased to 0.61% from 2041 to 2060, and decreased to 0.67%) from 2081 to 2100. Under SSP370, suitable habitat increased to 1.19% from 2021 to 2040, increased to 1.17% from 2061 to 2080, decreased to 0.26% from 2041 to 2060, and decreased to 0.43% from 2081 to 2100. A future decrease in suitable habitat for crocodile lizards was predicted under the highest radiative forcing scenario (SSP585), which would occur if temperatures continue to rise. Therefore, the average suitable habitat for *S. crocodilurus* would decrease more from 2021 to 2100 than the value predicted under the other three climate scenarios. In particular, high-quality habitat is expected to decrease to 0.95% from 2021 to 2040, 0.29% from 2041 to 2060 and 0.16% from 2061 to 2080. The proportion of high-quality habitats is expected to rebound briefly from 2081 to 2100, rising to 0.24% (Figure 5).

### 3.4. Dispersal Scenario Analysis

The model predictions showed that under the four climate models, the areas occupied by crocodile lizards are mainly restricted to several counties around Laibin city. There were also scattered habitats in Jinxiu Yao Autonomous County, Guiping City, Babu district of Hezhou City, Luokeng town, and Quảng Ninh province of Vietnam. The number of cells in unoccupied habitats is expected to decrease dramatically from SSP126 to SSP585.

A comparison of the projected distribution of crocodile lizards with their initial distribution for the year 2100 indicates that their distribution is always constrained under all the dispersal scenarios except for unlimited dispersal (Table 3). Under the unconstrained dispersal pattern, the crocodile lizards occupied more habitat cells under the low effective radiative forcing scenarios (SSP126 and SSP245) than their initial distribution. In the no-diffusion pattern, the habitat available for crocodile lizards decreased to zero with increasing values of effective radiative forcing (SSP370 and SSP585). In addition, it is worth mentioning that the results obtained from the no-diffusion and limited dispersal scenarios indicated that all crocodile lizards will lose their habitat when the effective radiative forcing reaches its maximum value (SSP585).

### 3.5. Conservation Units

**Figure 6:**
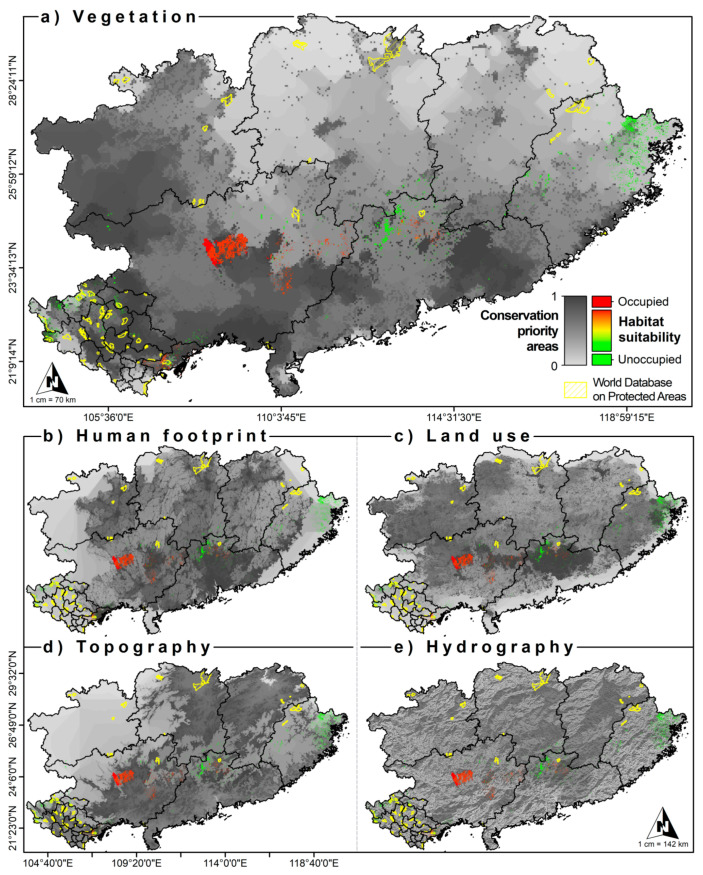
Conservation status for the *S. crocodilurus* species considering the World Database on Protected Areas (WDPAs). (**a**): priority conservation areas under vegetation; (**b**): priority conservation areas under human footprint; (**c**): priority conservation areas under land use; (**d**): priority conservation areas under topography; (**e**): priority conservation areas under hydrography.

The CAZ models obtained from the Zonation software allowed us to calculate priority areas for the conservation of our target species (Figure 6). Following the spatial distribution pattern in the current scenario, we can identify regions of importance to *S. crocodilurus*, acting as a possible priority and potential area for conserving the species. Some examples of this could be in Guangxi Dayao Mountain National Nature Reserve, Guangxi Daguishan Crocodile Lizard National Nature Reserve, Tay Yen Tu Nature Reserve (NR), BacGiang Province and Hai Ha District, Quang Ninh Province, or North Vietnam. The spatial distribution patterns for the LGM scenario revealed the previous importance of these areas to the species, their current importance, and their future significance.

## 4. Discussion

Based on Maxent and MigClim, we projected the potential suitable distribution range of *S. crocodilurus* under past, current, and future climatic scenarios, and the occupation of suitable habitat by crocodile lizards under different dispersal patterns. A comparison of current and past model distributions shows an increase in habitat since the LGM, specifically in east-central Guangxi Province in China. The predicted suitable distribution range for *S. crocodilurus* using occurrence records was consistent with its currently documented distribution, thereby verifying the predictive power of our models. *S. crocodilurus* is extremely vulnerable to future climate change, including global warming. Any future climate change (e.g., changes in precipitation and temperature) would reduce available habitat and negate the conservation effectiveness of the current nature reserve networks. Additionally, climate-induced changes to dispersal patterns will reduce the amount of habitat that crocodile lizards can occupy in the future. These findings should inform the dialogue about the role of climate change in the conservation of *S. crocodilurus* and thus have important implications for guiding future conservation planning.

### 4.1. Key Factors Affecting S. Crocodilurus Distributions

The most important factors explaining the current distribution of *S. crocodilurus* were related to temperature (contribution rate 34.2%) and precipitation (contribution rate 65.7%). This is not surprising given their importance to key physiological processes in reptiles [32,79,80]. Temperature increases associated with global warming may impact their ability to thermoregulate and maintain their preferred body temperature [81,82,83]. The two most common methods of temperature regulation used by animals are evaporative heat loss and dry heat exchange, and both are expected to be affected by climate warming [84], especially in reptiles. Failure to maintain body temperature within a critical range can lead to loss of physiological function and death [85,86]. For example, one consequence of global warming is the reduction of ‘hours of restriction’. This is the amount of time a lizard can spend active in its preferred body temperature range. Under global warming, if the hours of restriction cross a critical threshold lizards can no longer maintain basic physiological function to the degree necessary for reproduction. If this were to happen, the consequence would be local extinction [26].

In our study, the precipitation of the wettest month had the greatest effect on the crocodile lizards. There is a strong correlation between precipitation and high tempera-tures [87]. China and Vietnam are located in subtropical and tropical regions with simul-taneous hot and rainy seasons and high precipitation during the summer months [88,89]. The crocodile lizard breeding and mating season is from June to October and they usually mate in backwater ponds or streams [54]. Humid areas with high precipitation provides abundant food for crocodile lizards, which has an important positive impact on the breeding season [87,90]. Chinese crocodile lizards with small geographic ranges, large body size, high habitat specialization, and living in high precipitation areas are vulnerable to extinction [90]. Contemporary precipitation patterns can be used as a reflection of historical land-use changes [87]. Crocodile lizards are habitat specialists requiring high quality habitats [53,64,91]. Their habitat is dominated by broad-leaved forests with a veg-etation cover of >80% [65], which effectively blocks out UV rays and creates a microclimate with relatively stable temperatures. Geographic range size interacted significantly with precipitation and habitat specialization, indicating that they could increase the likelihood of extinction risk once range-restricted [90]. At the same time, increasing temperatures will also have a dramatic impact on future precipitation patterns because precipitation intervals will increase, as will the frequency of extreme precipitation events, increased precipitation at high latitudes, and decreased precipitation over most subtropical regions [92]. Changing precipitation patterns have led to larger and more frequent flooding events which could also impact *S. crocodilurus* [93].

*S. crocodilurus* is found in rocky streams that are part of typical karst landforms [48,49]. Karst landforms often have distinct features [94,95]. Owing to its carbonate nature, karst landscapes are generally characterized by thin surface soil, high soil infiltration capacity, and complex topography [95]. Vegetation in karst areas can use water from soils and ephemeral karst zones and may alter their water use strategies in the wet and dry seasons, resulting in plants occupying different landscape positions [96,97]. At the same time, thermal changes caused by daytime insolation and nighttime radiative cooling generate hydrostatic pressure gradients that cause rising or falling winds on the slopes, and this flow also leads to confluence and topographic precipitation [98]. Climate change and human activities affect the complex ecohydrological status of karst watersheds, which are more sensitive to changes in evapotranspiration than non-karst watersheds, and suggest that karst ecosystems may be subject to greater degradation pressures under future climate change scenarios [99,100].

Crocodile lizards usually prefer to perch on a branch above a backwater pond, dropping into the water as soon as they sense a threat [47,65]. This vegetation cover not only helps create a suitable microclimate but also likely reduces predation risk. The well-structured vegetation and distinctive karst topography where they occur can store large amounts of water during periods of abundant precipitation to counter the risk of drying out streams during dry periods. When extreme temperatures are encountered, the vegetation in the crocodile lizard’s habitat forms a natural temperature shield, allowing the lizard to survive in times of crisis [47,65]. Nevertheless, in most altered reserve habitats, the natural broadleaf forest has been gradually cut down for sale and replaced with more profitable trees, such as *Illicium verum* and tea shrubs. During this process, the ground vegetation is removed, and the soil is fertilized [49]. When this occurs, the vegetation that previously retained water and stabilized the ground disappears and the stream floods in the rainy season and dries up in the dry season, leaving a habitat no longer suitable for crocodile lizards [49].

### 4.2. Predicted Habitat Suitability

Global climate change is affecting the distribution of ectotherms and may be the cause of a number of conservation issues, such as the dramatic displacement of suitable climatic space for species, leading to a significant reduction in habitable sites and threatening the existence of many of them [101]. The results of the ENMs and MigClim model were consistent, with suitable habitats for crocodile lizards showing an overall trend of decreasing with increasing temperature. Importantly, under the highest radiative forcing scenario (SSP585), all habitat is completely lost between 2081 and 2100 (reduction rate 100%). Huang et al. [102] showed that Dayao Mountain may be an ancient habitat refuge and that Guangxi and Luokeng might have been the source populations for an initial population expansion. Both the LGM and the current findings highlight the importance of Luokeng for crocodile lizards, and that it may also have been an important historical refuge for crocodile lizards. The results of warm and particularly dry conditions may include unsustainable declines in replenishment, particularly in parts of a species range where heat/humidity conditions have become marginal [22].

Crocodile lizards feed on a wider prey spectrum including oligochaete worms, inver-tebrate larvae, cockroaches, crickets, and aquatic invertebrates such as shrimps. They also consume small vertebrates, such as fishes, frogs, tadpoles and small lizards [55,103,104,105,106,107]. Extreme hydrological events caused by climate change can have drastic impacts on stream invertebrate community assembly, and these are likely to become even more profound in the future as high and low-flow spells are expected to occur more frequently, not allowing time for communities to recover [105]. Although common species should be less prone to regional extinction and have better chances of recolonization after disturbance [106], they may also be locally more affected by stressors than rare species [107]. Therefore, changes in the abundance, range, activity, and reproductive cycles of these animals may have an impact on the main food source for crocodile lizards.

Most animal species may have at least a limited dispersal capacity and may not be able to colonize all their climatically suitable areas if other habitat requirements are not met [108]. Complete and zero-bound migration assumptions are two extreme possibilities, with future range changes likely to fall somewhere in between [108,109]. In the MigClim model, under the infinite diffusion mode, the suitable habitat of crocodile lizards first increases and then decreases. Under the no-diffusion and barrier diffusion modes, the suitable habitat of crocodile lizards decreased sharply, with a maximum reduction of 100%. Crocodile lizards are diurnal viviparous lizards, and during the active season, they are usually found perched on branches during the day, or hiding in the water with their heads above the surface, remaining sedentary for long periods and moving slowly [54]. At night, they sleep on perches or hide in burrows, where they are not easily awakened [54]. Their specialized behavior and strong site fidelity undoubtedly increase their risk of extinction in the face of rapid climate change scenarios because they have limited dispersal capacity. At the same time, without conservation intervention such as captive breeding, the decline of the population will only accelerate rather than decrease due to poaching and large-scale human disturbance to their habitat.

### 4.3. Conservation Units

The overlapping of the CAZ output models with the habitat suitability model made it possible to identify areas of importance for the development of management and conservation plans. In this sense, considering the vegetation (Figure 6a), a high spatial overlap was observed between the maximum values of habitat suitability and the maximum values obtained from CAZ. The vegetation variable is the result of the combination of topographic (Figure 6d) and hydrological (Figure 6e) landscape variables. However, they are also defined by anthropic actions (Figure 6b) and their effects on ecosystems (Figure 6c). Vegetation, topography, and hydrological conditions influence the growth, development, reproduction, and survival of the crocodile lizard [51,52,53,65], while human land use and harvesting directly influence wild populations and the quality of remaining habitat within the crocodile lizard’s range [49]. All these variables, by themselves or in combination, show a strong effect on the spatial distribution patterns of crocodile lizards. Despite the high overlap of the different CAZs models with the habitat suitability model, the number of the World Database on Protected Areas (WDPAs) is not spatially representative. This spatial limitation, both in amplitude and in heterogeneity, could not only affect this species but also other elements of regional biodiversity.

Overall, it appears that climate change is already ‘quietly’ affecting the survival of the crocodile lizard, and this is being compounded by increasing habitat fragmentation [47,65]. Our study, therefore, provides an important reference for existing crocodile lizard reserves to respond to, especially the existing reserves of crocodile lizards in Dayao Mountain, Luokeng, China, and Quảng Ninh Province, Vietnam, which need to strengthen existing protections to protect lizards and their habitats to ensure the long-term survival of core populations.

## 5. Conclusions

Our results indicate that climate change will have a significant impact on existing crocodile lizard habitats within the next 100 years and that the effects will be catastrophic. Temperature and precipitation are the two most important factors affecting the distribution of crocodile lizards. When temperatures increase, the suitable habitat for crocodile lizards decreases dramatically, especially under the highest radiative forcing scenario (SSP585), with little suitable habitat for crocodile lizards remaining by 2100. The number of habitat cells occupied by crocodile lizards decreases to zero with increasing temperature in both no-migration and obstructed migration dispersal scenarios. We believe that our modeling approach can provide a clear understanding of the distribution of habitat for the target species, identify potential habitat causes in other areas, and inform the conservation and management of existing habitats for the crocodile lizard.

## Figures and Tables

**Table 1:** Percentage contributions and permutation importance of the bioclimatic variables included in the Maxent algorithm for crocodile lizards.

Variable	Description	Contribution (%)
Bio13	Precipitation of Wettest Month	37.1
Bio19	Precipitation of Coldest Quarter	17.9
Bio4	Temperature Seasonality	14.3
Bio17	Precipitation of Driest Quarter	10.7
Bio7	Temperature Annual Range	10.4
Bio10	Mean Temperature of Warmest Quarter	9.5

**Table 2:** Parameter setting for MigClim.

Parameter	Description	Parameter Settings
rcThreshold	Habitat suitability data	500
envChgSteps	Number of the environmental change step	5
dispSteps	Number of the dispersal step	20
iniMatAge	Initial maturity age of newly colonized units	1
propaguleProd	Reproductive production potential of new colonization units over time	c(0.01 0.08 0.5 0.92)
lddFreq	the probability for an occupied cell to produce a long distance dispersal event	0.1
lddMinDist	Minimum distance for long distance dispersal	6
lddMaxDist	Maximum distance for long distance dispersal	15
replicateNb	Number of simulations repeated	5

**Table 3:** Cells occupied by crocodile lizard under different dispersal scenarios.

Climate Scenario	Unlimited Dispersal	No Dispersal	Limited Dispersal
Initial	4929	4929	4929
SSP126	6524	75	319
SSP245	5472	69	285
SSP370	3026	0	2
SSP585	1029	0	0

## Data Availability

All data are available from the Dryad. https://doi.org/10.5061/dryad.905qfttp5 (accessed on 9 August 2022).
